# A retrospective study of extracolonic, non-endometrial cancer in Swedish Lynch syndrome families

**DOI:** 10.1186/s13053-018-0098-9

**Published:** 2018-10-23

**Authors:** Masoud Karimi, Jenny von Salomé, Christos Aravidis, Gustav Silander, Marie Stenmark Askmalm, Isabelle Henriksson, Samuel Gebre-Medhin, Jan-Erik Frödin, Erik Björck, Kristina Lagerstedt-Robinson, Annika Lindblom, Emma Tham

**Affiliations:** 1 Department of Oncology, Karolinska University Hospital, Stockholm, Sweden; 2 Department of Clinical Genetics, Karolinska University Hospital, Solna, 171 76 Stockholm, Sweden; 3 Department of Molecular Medicine and Surgery, Karolinska Institutet, Stockholm, Sweden; 4 Department of Clinical Genetics, Akademiska University Hospital, Uppsala, Sweden; 5 Department of Clinical Genetics, Norrlands University Hospital, Umeå, Sweden; 6 Department of Clinical Genetics, Linköpings University Hospital, Linköping, Sweden; 7 Division of Clinical Genetics, Department of Laboratory Medicine, Lund University, Lund, Sweden; 8 Department of Clinical Genetics, Office for Medical Services, Division of Laboratory Medicine, Lund, Sweden

**Keywords:** Lynch syndrome, MMR genes, Tumour spectrum, Extracolonic

## Abstract

**Background:**

Lynch Syndrome is an autosomal dominant cancer syndrome caused by pathogenic germ-line variants in one of the DNA-mismatch-repair (MMR) genes *MLH1, MSH2, MSH6* or *PMS2*. Carriers are predisposed to colorectal and endometrial cancer, but also other cancer types. The purpose of this retrospective study was to characterize the tumour spectrum of the Swedish Lynch syndrome families.

**Methods:**

Data were obtained from genetically verified 235 Lynch families from five of the six health care regions in Sweden. The material was stratified for gender, primary cancer, age and mutated gene and the relative proportions of specific cancer types were compared to those in the general population.

**Results:**

A total of 1053 family members had 1493 cancer diagnoses of which 1011 were colorectal or endometrial cancer. Individuals with pathogenic variants in *MLH1* and *MSH2* comprised 78% of the cohort. Among the 482 non-colorectal/non-endometrial cancer diagnoses, *MSH2* carriers demonstrated a significantly increased proportion of urinary tract, gastric, small bowel, ovarian and non-melanoma skin cancer compared to the normal population. *MLH1* carriers had an elevated proportion of gastrointestinal cancers (gastric, small bowel, pancreas), while *MSH6* carriers had more ovarian cancer than expected. Gastric cancer was predominantly noted in older generations.

**Conclusion:**

Lynch syndrome confers an increased risk for multiple cancers other than colorectal and endometrial cancer. The proportions of other cancers vary between different MMR genes, with highest frequency in *MSH2*-carriers. Gender and age also affect the tumour spectrum, demonstrating the importance of additional environmental and constitutional parameters in determining the predisposition for different cancer types.

## Background

Lynch syndrome (LS) is an inherited autosomal dominant condition predisposing mainly to colorectal and endometrial cancer [1]. In addition, individuals with LS are at increased risk for developing malignancies other than colorectal and endometrial cancer, mostly cancer of the gastrointestinal tract, but also ovarian, urothelial and brain tumours have been reported to occur more often than in the general population [2–5]. LS is caused by pathogenic germ-line variants in DNA-Mismatch-Repair (MMR) genes, *MLH1*, *MSH2*, *MSH6* or *PMS2* [6] and the tumour spectrum is influenced mostly by the affected gene and gender [4, 5, 7]. Most pathogenic variants (80%) are reported in *MLH1* and *MSH2*, which might reflect differences in expression between different pathogenic MMR variants, such as later age at onset of disease and reduced penetrance suggested in *MSH6* and *PMS2* [1, 2, 7, 8].

LS should be suspected in patients with an age onset of colorectal cancer before 50 years, proximal localisation of colorectal cancer, high DNA microsatellite instability in histological specimen of colorectal cancer, multiple primary meta/synchronous polyps/tumours in colon, rectum and endometrium, endometrial cancer before 50 years of age, or a familial clustering of cancer diagnoses, using criteria such as Amsterdam II or Bethesda guidelines [9, 10]. Surveillance for colorectal cancer has been shown to increase survival in LS [11]. It is not yet clear if surveillance for other cancer types associated with LS would be beneficial in all patients. More knowledge about the phenotypical manifestation of different pathogenic variants in LS is required to improve the surveillance programs for different LS families. We therefore characterised the spectrum of tumours in the Swedish Lynch syndrome families and calculated the relative proportion of non-colorectal/non-endometrial cancers to further support their association to Lynch syndrome.

## Methods

The study was approved by the Regional Ethical Review Board in Stockholm, Sweden. In Sweden, patients with a family history suggestive of LS are generally referred for genetic counselling to a department of clinical genetics at six university hospitals that provide regional genetic services with family investigations, genetic testing and recommendations for surveillance. For our study, five of six nationwide genetic centres in Stockholm, Uppsala, Umeå, Linköping and Lund (covering 83% of Swedish population, i.e. 8.3 million individuals) agreed to participate, providing us with anonymous full pedigree information. The information regarding cancer diagnosis, age at onset, or date of death were confirmed by medical reports or death certificates when available with the written consent from the affected relative, or (if deceased) from the closest relatives. All pedigrees harboured at least 3 consecutive generations and contained information about gender, type of gene variant, birth date, age at cancer diagnosis, cancer according to the ICD7 classification, tumour site and age at death and the status of pedigrees has been updated as of December 2014.

Patients with early onset LS spectrum early onset cancer, synchronous or metachronous cancer, or Amsterdam II and/or Bethesda criteria fulfilled were genetically tested for LS. Genetic screening of the affected family members was performed in most cases using mainly Sanger DNA sequencing or otherwise massive parallel sequencing. The sequencing analyses were combined with multiplex ligation-dependent probe amplification (MLPA, P003 and P072; MRC-Holland, Amsterdam, The Netherlands) for the detection of large deletions or duplications.

### Statistics

Statistical analyses included family members with a proven pathogenic germ-line variant, obligate carriers, individuals with a 50% risk of having a pathogenic variant, or combinations thereof. Obligate carrier status was allocated to members due to their position in the pedigree in relation to relatives with known pathogenic variants or other obligate carriers. First degree relatives to proven or obligate carriers who had not been tested for the familial variant were assigned a 50% carrier probability. These 265 individuals with cancer increased the number of tumours in the analysis, but due to a potential risk of error as their genetic status was unknown, we redid the analysis only including those individuals with known genetic status with similar results (data not shown).

Analysis of the relative proportions of cancer diagnoses was performed as previously described [12]. The age at cancer diagnosis was known, but we did not have data on the year of birth or diagnosis of cancers in our observed data and could thus not calculate cumulative incidence. Therefore, the tumour distribution in the relatives of index patients is compared with the cancer distribution in the Swedish population at two time points, 1970 and 2010 (Swedish Cancer Registry). The population distribution of cancer was weighted by the age and sex of cases in the data (relatives to index cases). Cases where age or sex was missing were assumed to have the same age and sex distribution as cases where age and sex were known. We analysed each gene separately as well as each gender in the entire cohort.

## Results

In total, we obtained pedigrees from 235 families with Lynch syndrome *(MLH1 n* = 97, *MSH2 n* = 87, *MSH6 n* = 37 and *PMS2 n =* 14). In 1053 family members, at least one cancer diagnosis was identified (Table 1). Of these, 445 (42%) individuals had a proven pathogenic variant, 343 (33%) were obligate carriers and 265 (25%) individuals were assigned a 50% carrier probability. Table 1 summarizes the clinical characteristics and genetic status of the patients. In total, 1493 cancer diagnoses were found in our study cohort, of which 90% were verified using the cancer registry or medical records. Information on age at cancer diagnosis was available in 90% of patients. A total of 647 first-time colorectal cancers (if metachronous cancers were included the total was 795) and 203 cases of first-time endometrial cancer (216 including metachronous) were registered, corresponding to 43% and 14% of all reported cancer cases in the cohort, respectively (Table 2). Colorectal cancer represented 64% of all cancer in men and in 36% in women, while endometrial cancer represented 28% of all cancer in women (Table 2). A total of 482 cancers were non-colorectal, non-endometrial cancer. To calculate the relative proportion of these less common cancer diagnoses in the study cohort, all cases of colorectal and endometrial cancer were excluded from further analysis.

**Table 1:** Lynch syndrome family members distributed by gene and gender

	Male	Female	Total
	n (%)	n (%)	n (%)
*MLH1*	231 (22)	201 (19)	432 (41)
*MSH2*	194 (18)	195 (19)	389 (37)
*MSH6*	69 (7)	122 (12)	191 (18)
*PMS2*	25 (2)	16 (2)	41 (4)
Total	519 (49)	534 (51)	1053 (100)

**Table 2:** Number of first primary cancers, mean age at onset and range in Swedish Lynch family members

	CRC	%	Mean age	ENDO	%	Mean age	OV	%	Mean age	GC	%	Mean age	SmB	%	Mean age	UTC	%	Mean age	Skin	%	Mean age
MLH1, M	181	78%	47 (19–80)	NA	NA	NA	NA	NA	NA	15	6%	61 (19–92)	3	1%	62 (58–64)	6	3%	54 (40–66)	2	1%	64 (55–72)
MLH1, F	130	65%	49 (22–82)	67	33%	52 (36–80)	8	4%	50 (28–69)	11	5%	61 (38–84)	4	2%	56 (37–65)	10	5%	69 (51–80)	10	8%	64 (48–78)
MSH2, M	135	70%	50 (17–84)	NA	NA	NA	NA		NA	17	9%	63 (37–80)	5	3%	55 (48–74)	21	11%	58 (36–82)	10	7%	53 (40–65)
MSH2, F	86	44%	49 (23–83)	86	44%	50 (30–76)	20	10%	47 (35–80)	13	7%	56 (38–70)	3	2%	55 (53–57)	26	13%	57 (42–83)	14	16%	68 (54–81)
MSH6, M	40	58%	59 (33–82	NA	NA	NA	NA		NA	4	6%	42 (32–51)	NA	NA	NA	6	9%	66 (55–84)	1	3%	74 (NA)
MSH6, F	48	39%	58 (29–82)	47	39%	57 (41–80)	14	11%	53(40–75)	4	3%	59 (32–77)	NA	NA	NA	4	3%	65 (51–75)	NA	NA	NA
PMS2, M	18	72%	59 (37–83)	NA	NA	NA	NA		NA	2	8%	67 (56–77)	NA	NA	NA	1	4%	69 (NA)	NA	NA	NA
PMS2, F	9	56%	60 (23–93)	3	19%	59 (58–61)	1	6%	22 (NA)	1	6%	80 (NA)	NA	NA	NA	1	6%	62 (NA)	NA	NA	NA
All male	374	72%	50 (17–84)	NA	NA	NA	NA		NA	38	7%	62 (19–92)	8	2%	57 (48–74)	34	7%	58 (36–84)	13	3%	56 (40–72)
All female	273	51%	51 (22–93)	203	38%	53 (30–80)	43	8%	49 (22–80)	29	5%	59 (32–84)	7	1%	56 (37–65)	41	8%	61 (42–83)	24	9%	66 (40–81)
Total	1294	123%	51 (17–93)	NA	NA	NA	NA	NA	NA	67	6%	60 (19–92)	15	1%	56 (37–74)	75	7%	60 (36–84)	37	3%	62 (40–81)

The percentages represent the fraction per gene (and for the affected gender for gynecological tumours). Note that values of mean age at onset and range are calculated only for members with confirmed age at diagnosis

Compared to the general population, individuals of both sexes in the cohort as a whole had a higher proportion of gastric cancer (Tables 3 and 4). Gastric cancer was more frequent in male than female mutation carriers and tended to be present in older generations as only 6/67 cases in the cohort were born after 1940 (Table 2; data not shown). The relative proportion of small bowel cancer was also elevated in both men and women with Lynch syndrome and the mean age at onset was on average 4 years younger than for gastric cancer (Tables 2, 3 and 4). Females had an increased proportion of ovarian cancer, but also of non-melanoma skin cancer, the latter was not increased in men (Tables 3 and 4). While the proportion of urinary tract cancer was significantly elevated in females in the cohort, this was only true in males with a 100% probability of carrying a pathogenic MMR variant (data not shown). Prostate and breast cancer were common in male and female Lynch syndrome carriers respectively, but the proportion was not elevated compared to the general population, indeed, the proportion of breast cancer was lower than expected among our Lynch syndrome cohort).

**Table 3:** Observed cancer cases for the male Lynch syndrome cohort with 100% or 50% probability of *MMR* mutation (excluding colorectal and endometrial cancer)

Primary cancer	Observed number	Proportion [%]	LL 95%	UL 95%	Proportion [%] in Sweden 1970	Proportion [%] in Sweden 2010	Reference outside CI
Stomach	**38**	**18.36**	**13.04**	**23.67**	**8.84**	**1.76**	**above**
Prostate	38	18.36	13.04	23.67	10.82	26.47	No
Kidney and urinary tract excl prostate	34	16.43	11.59	21.74	13.05	8.75	No
Brain and nervous system	19	9.18	5.31	13.04	7.94	5.66	No
Skin excl melanoma	13	6.28	3.38	9.66	2.44	5.24	No
Pancreas	12	5.8	2.9	9.18	3.36	1.74	No
Malignant melanom	9	4.35	1.93	7.25	5.19	10.35	No
Blood and lymphatic tissue	9	4.35	1.93	7.25	12.53	12.3	below
Small bowel	**7**	**3.38**	**0.97**	**5.8**	**0.75**	**0.77**	**above**
Liver and biliary system	6	2.9	0.97	5.31	2.44	2.04	No
Lung and airways	5	2.42	0.48	4.83	11.38	5.51	below
Oesophagus	4	1.93	0.48	3.86	1.22	0.93	No
Head and neck	3	1.45	0	3.38	5.76	4.04	below
Bone and soft tissue	3	1.45	0	3.38	2.1	1.52	No
Testicle	2	0.97	0	2.42	3.43	6.54	below
Breast	1	0.48	0	1.45	0.33	0.28	No
Penis	1	0.48	0	1.45	0.66	0.37	No
Thyroid	1	0.48	0	1.45	1.13	1.37	No
Eye	0	0	0	0	0.58	0.44	below
Endocrine cancer	0	0	0	0	2.57	1.91	below
Unspecified location	0	0	0	0	3.45	2.01	below

The observed proportions adjusted for age and sex are compared to those of the general population in year 1970 and 2010 (ref National Board of Health and Welfare). If the observed confidence interval in the Lynch syndrome group did not overlap with the proportions in the general population, the reference is denoted as “above” (or “below”) the reference

*LL* Lower level of 95% confidence interval, *UL* upper level of 95% confidence interval

**Table 4:** Observed cancer cases for the female Lynch syndrome cohort with 100% or 50% probability of *MMR* mutation (excluding colorectal and endometrial cancer)

Primary cancer	Observed number	Proportion [%]	LL 95%	UL 95%	Proportion [%] in Sweden 1970	Proportion [%] in Sweden 2010	Reference outside CI
Breast	58	21.17	16.42	26.28	31.79	43.61	below
Ovary and Fallopian tube	**42**	**15.33**	**11.31**	**19.71**	**9.51**	**3.8**	**above**
Kidney/urinary tract excl prostate	**41**	**14.96**	**10.95**	**19.34**	**5.25**	**3.34**	**above**
Stomach	**29**	**10.58**	**6.93**	**14.23**	**4.47**	**1.11**	**above**
Skin excluding melanoma	**24**	**8.76**	**5.47**	**12.41**	**1.37**	**4.4**	**above**
Cervix	20	7.3	4.38	10.58	10.72	3.71	No
Brain and nervous system	12	4.38	2.19	6.93	4.58	4.5	No
Blood and lymphatic tissue	11	4.01	1.82	6.57	7.45	6.81	below
Small bowel	**8**	**2.92**	**1.09**	**5.11**	**0.38**	**0.4**	**above**
Liver and biliary system	7	2.55	0.73	4.74	2.87	1.34	No
Lung and airways	5	1.82	0.36	3.65	3.03	5.95	No
Pancreas	4	1.46	0.36	2.92	2.38	1.58	No
Malignant melanoma	4	1.46	0.36	2.92	3.8	8.63	below
Thyroid	3	1.09	0	2.55	2.01	2.59	No
Oesophagus	2	0.73	0	1.82	0.37	0.31	No
Bone and soft tissue	2	0.73	0	1.82	1.16	0.71	No
Head and neck	1	0.36	0	1.09	1.68	1.92	below
Endocrine cancer	1	0.36	0	1.09	2.44	2.09	below

The observed proportions adjusted for age and sex are compared to those of the general population in year 1970 and 2010 (ref National Board of Health and Welfare). If the observed confidence interval in the Lynch syndrome group did not overlap with the proportions in the general population, the reference is denoted as “above” the reference

*LL* Lower level of 95% confidence interval, *UL* upper level of 95% confidence interval

Of note, most of the extracolonic, non-endometrial malignancies occurred as single cases in the kindred. A few families demonstrated multiple individuals with the same cancer, e.g. in one family there were four cases of gastric cancer and two families had four cases of ovarian cancer. Breast cancer also clustered in a few families (Table 5).

As the spectrum of LS associated tumours is influenced by the genetic composition of the cohorts, we stratified the study population according to mutated MMR gene. We could not analyse *PMS2* carriers, as the number of cases with non-colorectal/non-endometrial cancers (*n* = 7) was too low for further analysis.

### MLH1

*MLH1* carriers had an elevated frequency of gastric, pancreas and small bowel but not of skin, urinary tract or ovarian cancer (Table 6). The mean age at diagnosis for all these cancers in our *MLH1* cohort was a few years younger than the average age in the population (Swedish Cancer Registry).

**Table 5:** Intrafamilial clustering of some common cancers in Swedish Lynch syndrome families

	1 case/family	2 case/family	3case/family	4 case/family	5 case/family
Gastric cancer	58 (87%)	5 (7.5%)	3 (4%)	1 (1.5%)	0
Brain tumour	25 (81%)	6 (19%)	0	0	0
Urinary tract cancer	61 (81%)	9 (12%)	5 (7%)	0	0
Ovarian cancer	37 (86%)	3 (7%)	1 (2%)	2 (5%)	0

Number of families with multiple cases of the specified tumour and percent of the total

### MSH2

Carriers of pathogenic variants in *MSH2* carriers had an elevated proportion of several cancers including urinary tract, gastric, non-melanoma skin, ovarian and small bowel cancer (Table 7). The *MSH2* carriers diagnosed with urinary tract and ovarian cancer showed a greater proportion with onset before age of 50 years in comparison with *MLH1* carriers (Table 9). In fact, 64% of ovarian cancers in the entire cohort were diagnosed before the age of 50 years. (Table 8) and 25% had an onset before the age of 40 years among all carriers with this especially true for *MSH2* carriers where 34% were diagnosed before 40 years (data not shown). Of note, the elevated proportion of non-melanoma skin cancer found in *MSH2* carriers (Table 7) was reflected in the female but not the male MMR carrier group (Tables 3 and 4).

**Table 6:** Observed cancer cases for the MLH1 cohort with 100% or 50% probability of *MMR* mutation (excluding colorectal and endometrial cancer)

Primary site	Observed number	Proportion [%]	LL 95%	UL 95%	Proportion [%] in Sweden 1970	Proportion [%] in Sweden 2010	Reference outside CI
Breast	28	17.72	12.03	24.05	16.61	23.02	No
Stomach	**26**	**16.46**	**10.76**	**22.15**	**6.31**	**1.4**	**above**
Kidney/urinary tract excl prostate	16	10.13	5.7	15.19	8.99	5.77	No
Skin excl melanoma	12	7.59	3.8	12.03	1.76	4.5	No
Pancreas	**11**	**6.96**	**3.16**	**11.39**	**2.66**	**1.6**	**above**
Brain and nervous system	10	6.33	2.53	10.13	6.61	5.34	No
Ovary and Fallopian tube	8	5.06	1.9	8.86	4.97	2.02	No
Small bowel	**7**	**4.43**	**1.27**	**7.59**	**0.57**	**0.57**	**above**
Liver and biliary system	6	3.8	1.27	6.96	2.52	1.65	No
Malignant melanoma	6	3.8	1.27	6.96	4.98	10.18	No
Cervix	5	3.16	0.63	6.33	5.79	2.08	No
Prostate	5	3.16	0.63	6.33	4.26	11.02	No
Head and neck	4	2.53	0.63	5.06	3.69	2.95	No
Lung and airways	4	2.53	0.63	5.06	6.79	5.46	below
Oesophagus	3	1.9	0	4.43	0.74	0.58	No
Blood and lymphatic tissue	3	1.9	0	4.43	10.02	9.72	below
Bone and soft tissue	2	1.27	0	3.16	1.75	1.17	No
Testicle	1	0.63	0	1.9	1.98	3.65	below
Thyroid	1	0.63	0	1.9	1.68	2.22	No
Female genital organ	0	0	0	0	0.54	0.33	below
Penis	0	0	0	0	0.32	0.18	below
Eye	0	0	0	0	0.48	0.35	below
Endocrine cancer	0	0	0	0	2.66	2.12	below
Unspecified location	0	0	0	0	3.32	2.14	below

The observed proportions adjusted for age and sex are compared to those of the general population in year 1970 and 2010 (ref National Board of Health and Welfare). If the observed confidence interval in the Lynch syndrome group did not overlap with the proportions in the general population, the reference is denoted as “above” the reference and is marked in bold

*LL* Lower level of 95% confidence interval, *UL* upper level of 95% confidence interval

### MSH6

In the group with pathogenic variants in *MSH6*, ovarian cancer was noted in around 11.5% versus 4% in the *MLH1* group (Tables 6 and 9). The proportion of gastric cancer in *MSH6* carriers was higher than normal in the general population but the difference was not statistically significant (Table 9). Of 8 cases of gastric cancer in the *MSH6* cohort, the age at diagnosis could be confirmed for 6 and 4 of these (67%) occurred before age of 50 years (Table 8).

**Table 7:** Observed cancer cases for the MSH2 cohort with 100% or 50% probability of *MMR* mutation (excluding colorectal and endometrial cancer)

Primary site	Observed number	Proportion [%]	LL 95%	UL 95%	Proportion [%] in Sweden 1970	Proportion [%] in Sweden 2010	Reference outside CI
Kidney/urinary tract excl prostate	**47**	**21.56**	**16.06**	**27.06**	**8.93**	**5.87**	**above**
Stomach	**30**	**13.76**	**9.17**	**18.35**	**6.44**	**1.41**	**above**
Skin excl melanoma	**24**	**11.01**	**6.88**	**15.14**	**1.87**	**4.54**	**above**
Ovary and Fallopian tube	**20**	**9.17**	**5.5**	**13.3**	**4.86**	**1.92**	**above**
Prostate	17	7.8	4.59	11.47	5.26	13.07	No
Breast	15	6.88	3.67	10.55	16.64	23.03	below
Brain and nervous system	12	5.5	2.75	8.72	6.19	5.13	No
Cervix	11	5.05	2.29	8.26	5.86	2.08	No
Small bowel	**8**	**3.67**	**1.38**	**6.42**	**0.54**	**0.56**	**above**
Blood and lymphatic tissue	8	3.67	1.38	6.42	9.98	9.29	below
Malignant melanoma	6	2.75	0.92	5.05	4.51	9.58	No
Pancreas	4	1.83	0.46	3.67	2.81	1.63	No
Lung and airways	4	1.83	0.46	3.67	7.12	5.49	below
Liver and biliary system	3	1.38	0	3.21	2.57	1.65	No
Thyroid	3	1.38	0	3.21	1.63	2.02	No
Oesophagus	2	0.92	0	2.29	0.76	0.62	No
Bone and soft tissue	2	0.92	0	2.29	1.59	1.07	No
Testicle	1	0.46	0	1.38	1.63	3.12	below
Penis	1	0.46	0	1.38	0.34	0.18	No
Head and neck	0	0	0	0	3.67	2.97	below
Female genital organ	0	0	0	0	0.48	0.3	below
Eye	0	0	0	0	0.46	0.34	below
Endocrine cancer	0	0	0	0	2.5	1.99	below
Unspecified location	0	0	0	0	3.34	2.13	below

The observed proportions adjusted for age and sex are compared to those of the general population in year 1970 and 2010 (ref National Board of Health and Welfare). If the observed confidence interval in the Lynch syndrome group did not overlap with the proportions in the general population, the reference is denoted as “above” the reference and is marked in bold

*LL* Lower level of 95% confidence interval, *UL* upper level of 95% confidence interval

**Table 8:** Number and proportion (%) of Swedish Lynch syndrome family members with onset of primary cancer < 50 years age in relation to gender and MMR gene mutation

	CRC No. (%)	EC No (%)	OVC No. (%)	UTC No. (%)	GC No. (%)	SB No. (%)	NMS No. (%)
Male	203 (54)	NA	NA	8 (27)	7 (22)	3 (38)	5 (42)
Female	139 (51)	92 (45)	28 (64)	9 (26)	7 (27)	2 (25)	1 (6)
MLH1	192 (62)	33 (50)	5 (63)	3 (19)	5 (25)	2 (25)	1 (13)
MSH2	119 (54)	50 (59)	16 (80)	14 (34)	6 (20)	3(39)	5 (27)
MSH6	23 (26)	9 (19)	6 (42)	–	4 (67)	–	–
PMS2	8 (30)	–	–	–	–	–	–

Calculation based on the group of patients with verified age at diagnosis and not on the entire cohort

*CRC* Colorectal cancer, *EC* Endometrial cancer, *OC* Ovarian cancer, *UTC* Urinary tract cancer (excluding prostate cancer), *GC* Gastric cancer, *SB* Small bowel cancer, *NMS* non-melanoma skin cancer

The number of affected individuals with small bowel cancer (*n* = 0), skin cancer (*n* = 1) and urinary tract cancer (*n* = 10) was too limited for analysis in the *MSH6* group.

## Discussion

This is the first retrospective analysis of the phenotype of the Swedish LS families ascertained by the Departments of Clinical Genetics across the nation. The spectrum of pathogenic MMR variants in Swedish families has been previously reported [13] and this study further explores the tumour spectrum of the Swedish Lynch syndrome families.

It is well established that carriers of heterozygous pathogenic variants in *MLH1, MSH2, MSH6* and *PMS2* have an increased risk of colorectal and endometrial cancer and that the four MMR genes demonstrate different penetrance and expressivity [2–5]. In order to determine the relative frequency of the less common cancers in the Swedish LS population, we stratified our cohort by mutated MMR gene and gender and excluded colorectal cancer and endometrial cancer from the statistical analysis.

### Gastric cancer

Both male and female carriers overall and both *MLH1* and *MSH2*-carriers had an increased proportion of gastric cancer, which was not seen in *MSH6*-carriers. A recent prospective study including 3119 Lynch syndrome patients demonstrated a cumulative risk for gastric cancer of 7.1/7.7% in *MLH1/MSH2* carriers and 5.3% in *MSH6* carriers [7]. Of interest, other studies have shown a preponderance of male gastric cancer [3–5] which was also the case among our Lynch syndrome cases. According to the Swedish cancer registry, a clear decrease in annual incidence and relative proportion for gastric cancer in the general population has been observed: from 5,4% in 1970 to 1,22% in 2010. Barrow et al. showed a decreasing incidence of gastric cancer in Lynch families and fewer than 10% of the Lynch syndrome carriers born after 1935 developed gastric cancer [3]. A similar finding could also be noted in our cohort with only 6/67 cases with gastric cancer born after 1940. This relatively low incidence of gastric cancer in later Lynch generations raises the issue of the value of screening gastroscopy in Lynch families, especially as most cases occurred as single sporadic cases within families. The clinical benefit of screening Lynch patients with gastroscopy probably exists for a very limited group, but the yield is likely too small to be cost-effective.

### Small bowel cancer

Small bowel cancer is a rarity, representing 0.5% of cancer cases in Sweden 2010, while the cumulative lifetime risk in the LS group has been estimated to be between 0.6–7% [4, 5]. The significantly increased proportion for of small bowel cancer in our LS in our cohort was evenly distributed between both sexes with similar risks for *MLH1* and *MSH2* carriers. No cases of small bowel cancer were observed in *MSH6* carriers. A recently published prospective Dutch study examined the eventual benefit of capsule endoscopy (the recommended surveillance procedure) in 200 asymptomatic LS family members. No cases of small bowel cancer could be detected in the study group during the 2-year surveillance [14]. Recently updated guidelines from the Mallorca group (2013) do not recommend any screening for small bowel cancer [15]. Based on these studies, our data and the rarity of this diagnosis, a routine screening for detection of small bowel cancer is questionable.

### Ovarian cancer

The cumulative lifetime risk for ovarian cancer in LS is reported to be between 7 and 24% up to age 70 years and varies between genotypes, with most older studies reporting the highest risks in *MSH2* and *MLH1* carriers, as the number of families with pathogenic variants in *MSH6* have been too low in most studies for conclusive results [4, 16]. Newer studies including *MSH6* carriers indicate a 10–13% cumulative risk for ovarian cancer, comparable to that of *MSH2* carriers and perhaps higher than the risk for *MLH1* carriers [5, 7]. This was also seen in our cohort with the *MSH2* and *MSH6* carriers demonstrating an increased frequency of ovarian cancer, while the *MLH1* carriers did not. A striking observation in our study was the high proportion of ovarian cancer before the age of 50 years in mutation carriers: 80% for *MSH2,* 63% for *MLH1* and 42% for *MSH6* (Table 9). Similar results have been noted by Helder-Woolderink and co-workers in their systematic review of ovarian cancer in LS family members where 29% of the cases had an onset before the age of 35 years [17].

**Table 9:** Observed cancer cases for the MSH6 cohort with 100% or 50% probability of *MMR* mutation (excluding colorectal and endometrial cancer)

Primary cancer	Observed number	Proportion [%]	LL 95%	UL 95%	Proportion [%] in Sweden 1970	Proportion [%] in Sweden 2010	Reference outside CI
Breast	16	17.78	10	25.56	21.31	29.43	No
Ovary and Fallopian tube	**14**	**15.56**	**8.89**	**23.33**	**6.4**	**2.6**	**above**
Prostate	13	14.44	7.78	22.22	5.5	11.58	No
Kidney/urinary tract excl prostate	10	11.11	5.56	17.78	8.24	5.57	No
Stomach	8	8.89	3.33	15.56	6.74	1.38	No
Brain and nervous system	8	8.89	3.33	15.56	4.88	4.16	No
Blood and lymphatic tissue	7	7.78	2.22	13.33	8.66	8.21	No
Cervix	4	4.44	1.11	8.89	5.93	1.79	No
Liver and biliary system	3	3.33	0	7.78	3.14	1.71	No
Pancreas	2	2.22	0	5.56	3.23	1.85	No
Lung and airways	2	2.22	0	5.56	6.49	7	below
Skin excl melanom	1	1.11	0	3.33	1.96	5.44	No
Endocrine cancer	1	1.11	0	3.33	2.17	1.81	No
Bone and soft tissue	1	1.11	0	3.33	1.24	0.83	No
Head and neck	0	0	0	0	2.94	2.55	below
Oesophagus	0	0	0	0	0.75	0.54	below
Small bowel	0	0	0	0	0.5	0.56	below
female genital organ	0	0	0	0	0.72	0.46	below
Testicle	0	0	0	0	0.49	0.9	below
Penis	0	0	0	0	0.18	0.11	below
Malign melanom	0	0	0	0	3	7.23	below
Eye	0	0	0	0	0.47	0.34	below
Thyroid	0	0	0	0	1.35	1.59	below
Unspecified location	0	0	0	0	3.72	2.37	below

The observed proportions adjusted for age and sex are compared to those of the general population in year 1970 and 2010 (ref National Board of Health and Welfare). If the observed confidence interval in the Lynch syndrome group did not overlap with the proportions in the general population, the reference is denoted as “above” the reference and is marked in bold

*LL* Lower level of 95% confidence interval, *UL* upper level of 95% confidence interval

Screening for gynaecological cancer has not proven to be effective in detecting pre-malignant lesions [18–20], even though single individuals with precursor cystic lesions have been detected at an early stage [21]. Results from a recently published multicentre prospective study of surveillance performed on 1942 *MLH1*and *MSH2* carriers without previous cancer, also point to the unsatisfying efficacy of gynaecological screening as precursor lesions were seldomly found in the endometrium or ovaries [8]. At least two studies have shown benefit from prophylactic salpingo-oophorectomy; 0–0.006% of the operated women developed ovarian/peritoneal cancer compared to 3.7–5% in the non-operated group [22, 23]. Considering the uncertain benefit of gynaecological screening and lack of an existing consensus regarding the efficacy gynaecological surveillance, our finding of a high proportion of ovarian cancer with an onset before the age of 40 years when reproduction might not yet be completed adds further dilemma to ongoing discussions about surveillance and the timing of preventive salpingo-oophorectomy in women before menopause.

### Urinary tract cancer

In addition, females with Lynch syndrome as well as *MSH2* carriers had an increased proportion of urinary tract, the latter in line with other studies [2, 5, 7, 16, 24]. Regarding urinary tract cancer (including renal pelvis, urothelial and bladder cancer but excluding prostate cancer), other studies have indicated a cumulative risk of 2–12% up to 70 years of age overall, with the highest risk (7–28%) in men with pathogenic *MSH2* variants [7, 16, 24–26]. An interesting finding by Watson and co-workers (2008) was the observation of increased incidence rates of urinary tract cancer in Danish and Finnish LS families in comparison to LS families from the Netherlands and USA [16], suggesting geographical differences. In our study, we only noted an increased proportion of urinary tract cancer in female Lynch syndrome carriers in the whole group, whereas for male carriers, only those with proven pathogenic MMR variants had an increased frequency, suggesting that this discrepancy is likely due to the limited numbers of affected in the study, although a regional effect cannot be excluded. Both sexes showed a similar proportion with an age of onset before 50 years for urinary tract cancer (Table 9). Most urinary tract cancers occurred as isolated single cases within families as previously noted [25]. The increased proportion of urinary tract cancer raises questions about possible surveillance for this diagnosis in LS families. However, as of today no consensus exists regarding the benefit, appropriate procedures or intensity of surveillance programs [26, 27]. The Mallorca group in their update of guidelines 2013 for clinical management of LS does not recommend any surveillance for this cancer type except in clinical trials [15].

### Non-melanoma skin cancer

Interestingly, female Lynch syndrome carriers had an increased proportion of non-melanoma skin cancer, also evident in the *MSH2* carrier group. This is an interesting finding that needs corroboration in other studies. Of note, our diagnosis includes all malignant tumours – including sebaceous carcinoma, but not sebaceous adenoma which is known to be associated with a variant of Lynch syndrome called Muir-Torre syndrome [28]. The incidence of skin cancer, both melanoma and non-melanoma, is increasing in northern Europe and Sweden but this increase is for both sexes in general population. Our finding showing increased numbers for non-melanoma skin cancer in a sex-dependent way could partly mirror an altered lifestyle, a changing landscape of malignancies in the Lynch population or be the result of other yet undefined causes. A prospective study of subsequent cancers in LS patients suggested an increase in skin cancer with age, which would support this hypothesis, but the results were difficult to interpret as skin cancer may be underreported [29].

### Pancreatic cancer

There has been some controversy about including pancreatic cancer in the LS-associated cancer spectrum. Two prospective studies [29, 30] and one retrospective study [31] indicated that LS family members had an increased susceptibility for pancreatic cancer. In our cohort, pancreatic cancer showed a relative increase in *MLH1* carriers only, in line with the most recent prospective study showing a cumulative risk of 6.2% for pancreas cancer in *MLH1* carriers only [7]. This finding needs further validation in larger studies. Since no family in our cohort had more than one case of pancreatic cancer there is probably no value of screening for pancreatic cancer in families with Lynch syndrome.

### Prostate and breast cancer

Prostate and breast cancer were common in male and female Lynch syndrome carriers respectively, but the proportion was not elevated compared to the general population, indeed, the proportion of breast cancer was lower than expected among our Lynch syndrome cohort.

At present, neither prostate cancer nor breast cancer is considered part of the tumour spectrum in LS but a possible role for both cancers in LS is under debate. Previous studies evaluating the risk for breast cancer in LS have had conflicting results and only few have included all four MMR genes. In a recent study by Möller et al., the risk for breast cancer in patients with LS is not significantly increased, which is in line with our results [7]. One explanation to the lower than expected proportion of breast cancer in our cohort might be that the prevalence of Lynch syndrome related tumours is high reducing the relative contribution of breast cancer in our cohort. In addition, breast cancer is common in the general population and has a later age of onset compared to most LS associated cancers. Thus, in former generations when LS patients often died from their first cancer, breast cancer was not as common. For prostate cancer, the proportion in our cohort did not show any tendency towards higher values than the general population, a result that was unchanged even after stratifying the cohort for different MMR genes. An increased incidence of prostate cancer among LS patients has been suggested, but also here different studies present conflicting results. Möller et al. 2018 reports an increased incidence of prostate cancer in a prospective dataset of patients with *MSH2* pathogenic variants, with a later age at onset that other LS associated cancers [7]. As early detection of invasive colorectal cancer is associated with a very high survival today, patients are more likely to develop prostate cancer later in life, as opposed to former generations. With our retrospective design this might affect the results, given that the prevalence of prostate cancer is high in Sweden and a potential increased risk is likely to be modest and occur at an older age.

### Limitations and strengths

Our material is based on recruitment of patients with colon cancer and/or endometrial cancer, through the Swedish Departments of Oncogenetics and usually there is early onset of cancer or clustering of several cancer diagnoses in the family. This may have led to selection bias, e.g. cases with pathogenic MMR variants of low penetrance (most likely those with *MSH6* or *PMS2* variants) will be missed. Inaccessibility to older medical reports was another concern, preventing the verification of cancer diagnosis in around 10% of the older generations in our study cohort. The retrospective nature of our study and the paucity of individuals with pathogenic variants in *PMS2* and *MSH6* necessitated wide confidence intervals that may have led to an underestimation of cancer types in these two groups. In addition, in former generations LS patients often died from their first cancer, as opposed to today when most LS patients under surveillance survive their first as well as subsequent cancers and thus may develop other late-onset tumours not seen in previous generations. This may bias our results.

The study has the benefit of being almost nationwide covering a population of around 8.3 million (83% of country’s population). In addition, pedigrees were comparably vast, containing at least three consecutive generations. Furthermore, the size of our cohort is not negligible covering a total of 1053 LS patients with cancer. Another strength of our study was the confirmation of clinical data (i.e., cancer diagnoses and age at onset) in 90% of our LS cohort through the Swedish cancer registry.

## Conclusion

In summary, the tumour spectrum of Swedish Lynch syndrome patients overlaps with that of LS patients in other Western countries. In addition to the increased risk of colon and endometrial cancer, *MSH2* carriers develop multiple other cancers including gastric, urinary tract, ovarian, small bowel and non-melanoma skin cancer. In contrast, *MLH1* carriers show an increased proportion of gastrointestinal cancers and *MSH6* carriers of ovarian cancer. In addition, gender affected the tumour spectrum, with non-melanoma skin cancer noted in women only. The tumour spectrum also varies between genders and as over time, demonstrating the importance of not only genetic but also environmental factors in determining cancer predisposition. Our results may contribute to more accurate cancer risk estimations in Lynch syndrome patients thus providing better evidence upon which to base surveillance recommendations.
